# Synergistic biodegradation of polyethylene by experimentally evolved bacterial biofilms

**DOI:** 10.1093/ismejo/wraf223

**Published:** 2025-10-08

**Authors:** Shan Li, Jiajia Liu, Lei Su, Jingwen Qiu, Lianbing Lin, Ákos T Kovács, Yicen Lin

**Affiliations:** Faculty of Life Science and Technology, Kunming University of Science and Technology, Kunming 650500, Yunnan Province, China; Faculty of Life Science and Technology, Kunming University of Science and Technology, Kunming 650500, Yunnan Province, China; Faculty of Life Science and Technology, Kunming University of Science and Technology, Kunming 650500, Yunnan Province, China; Faculty of Life Science and Technology, Kunming University of Science and Technology, Kunming 650500, Yunnan Province, China; Faculty of Life Science and Technology, Kunming University of Science and Technology, Kunming 650500, Yunnan Province, China; Institute of Biology, Leiden University, Leiden 2333BE, The Netherlands; Faculty of Life Science and Technology, Kunming University of Science and Technology, Kunming 650500, Yunnan Province, China

**Keywords:** polyethylene biodegradation, evolution, bacterial biofilms, transcriptomics, extracellular polysaccharide

## Abstract

Polyethylene (PE), one of the most widely used synthetic polymers, presents significant environmental challenges due to its resistance to biodegradation. Its surface offers a unique ecological niche for microbial colonization and serves as a primary habitat for degrading microorganisms. Despite the pivotal role microbial communities play in plastic degradation, there has been limited research on constructing stable, interacting microbial consortia. In this study, we explored the potential of evolving bacterial biofilm communities to enhance PE degradation. Through long-term experimental evolution, six microbial populations underwent 40 selection cycles using PE as their sole carbon source. The resulting evolved communities formed robust, multi-species biofilms with enhanced degradation capabilities, outperforming their ancestral populations in biofilm production. *Stutzerimonas stutzeri* emerged as the dominant species, orchestrating a synergistic interaction with two other isolates through metabolic division of labor. (Meta)-transcriptomics analysis revealed that *Stutzerimonas* primarily contributed to the expression of enzymes involved in microbe-mediated degradation of PE, whereas the other community members were responsible for secreting extracellular polysaccharides, improving biofilm formation. This study highlights the potential of experimentally evolved microbial consortia to synergistically accelerate plastic biodegradation, offering promising strategies for environmental bioremediation.

## Graphical Abstract

**Graphical Abstract:**
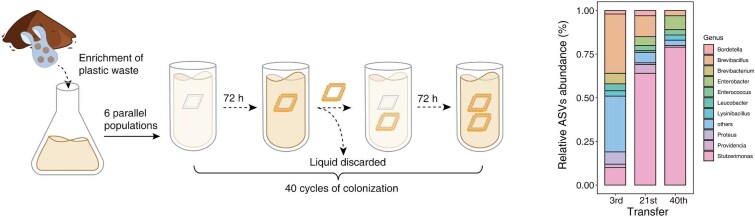

## Introduction

The durability and short lifespan of plastic products, such as polyethylene (PE), have led to the accumulation of plastic waste in the environment [1]. Once introduced, plastic debris creates a unique, unnatural habitat called the “plastisphere,” where microorganisms colonize and grow as multicellular aggregates within a self-produced matrix of extracellular polymeric substances (EPS), forming biofilms [2]. Bacterial biofilms on plastic debris differ from those in surrounding waters, showing lower diversity but a higher abundance of Proteobacteria [3, 4]. Although research on soil-based plastisphere communities is limited, previous studies suggest a distinct composition unlike those in the surrounding soil [5]. Furthermore, various plastics and their additives promote bacterial biofilm formation [6–8], underscoring the complex interactions between plastics and microorganisms when addressing environmental concerns.

Microorganisms are increasingly recognized as a promising solution towards reducing plastic pollution [9]. Recent reviews have explored microbial community composition in microplastic environments and their roles in plastic biodegradation [3, 10, 11]. Environmental plastic samples often host biofilms that contain significant sources of plastic-degrading bacteria [12]. For instance, a study identified key plastic-degrading microbes by comparing bacterial communities between microplastics and sediments [13]. The application of selective pressure can further enhance these microbes’ degradation capabilities, leading to communities that surpass naturally occurring ones. Consequently, enrichment methods using plastics as the sole carbon source are commonly employed to isolate plastic-degrading bacteria, such as *Bacillus* [9], *Pseudomonas* [14], *Rhodococcus* [12], and others [15].

However, reproducing such efficiencies with monocultures outside the laboratory is difficult. Unlike multispecies biofilms, monocultures lack communicative and metabolic complementarity and often suffer from metabolic overload during later degradation stages [16]. In contrast, naturally evolved biofilm communities overcome these limitations through metabolic division of labor, where distinct strains perform complementary functions [17]. Cross-feeding of metabolites between species enhances biodegradation efficiency, and spatial organization within biofilms facilitates these interactions [18, 19]. For example, initial plastic degradation often occurs within biofilms, with byproducts further broken down by surrounding bacterial communities, enabling complete plastic mineralization [20]. Thus, bacterial communities demonstrate superior environmental adaptability and degradation efficiency compared to monocultures. In a microbial community, keystone species are fundamental for maintaining community structure and function, and their absence may lead to dramatic shifts in community diversity and ecosystem performance [21]. Identifying and validating keystone species is therefore critical for the rational design of engineered microbial communities.

Despite growing recognition of bacterial communities in biodegradation, constructing stable, interacting communities remains a challenge. Several studies focus on enrichment methods, where pollutants serve as the sole carbon source, to construct degrading communities [22, 23]. Though effective, these methods often overlook the critical role of spatial organization that develops during long-term biofilm evolution. Experimental evolution of bacterial biofilms offers an alternative approach, promoting mutualism through repeated cycles of colonization and re-colonization [24]. In addition to directly degrading plastics, the enhancement of microbial biofilms aids in aggregating microplastics and removing larger particles from the environment. For example, Chan *et al.* [25] used experimental evolution to generate microplastic aggregates, which enhanced biofilm formation of *Pseudomonas aeruginosa* and concentrated microplastics to mitigate pollution. Evolved biofilm populations could rapidly diversify into distinct subpopulations that enhance overall fitness and productivity during infections [26]. Over the past decade, biofilms on plastic beads have provided an excellent model for studying biofilm evolution and diversification under experimental conditions [27, 28].

Building on our previous experimental evolution approach, we adapted a system for long-term bacterial evolution on plastic surface [29]. A stable, triple-species consortium isolated from an evolved population demonstrated superior PE degradation efficiency compared to monocultures. This enhanced performance was attributed to its increased maintenance capacity and higher extracellular matrix production of the consortium on PE plastic films, resulting from asymmetric cross-feeding and spatial partitioning of biofilm space. Overall, our findings highlight the potential of experimentally evolved bacterial consortia for enhancing plastic waste bioremediation.

## Materials and methods

### Establishment of experimental evolution

Naturally weathered discarded plastic bags were collected from the sediment of a lakeside environment in Dianchi, Kunming, Yunnan Province, China (102.7671°E, 24.8185°N). Plastic pieces (~2 × 4 cm) were prepared using sterile scissors and incubated in 100 ml of liquid carbon-free basal medium (LCFBM) supplemented with 0.01% yeast extract. The incubation was carried out in 300 ml flasks at 30°C, with shaking at 120 rpm, for 30 days. Following incubation, the cultures were vortexed and centrifuged to harvest the cells. The collected cells were resuspended in fresh LCFBM as starting cultures for further evolution experiment. LCFBM was prepared with deionized water, containing (per 1000 ml): 0.7 g KH_2_PO_4_, 0.7 g K_2_HPO_4_, 0.7 g MgSO_4_·7 H_2_O, 1.0 g NH_4_NO_3_, 0.005 g NaCl, 0.002 g FeSO_4_, 0.002 g ZnSO_4_, and 0.001 g MnSO_4_. Linear low-density polyethylene film (951-050, 30 μm thick) was obtained from SINOPEC Guangdong Maoming Company, China. According to the manufacturer’s specifications, this PE product contained no added catalysts or additives.

One milliliter of enriched culture (optical density at 600 nm (OD_600_) of 0.1) was inoculated into 14 ml of LCFBM. The culture was incubated in test tubes at 30°C with shaking at 120 rpm, allowing biofilm formation on floating PE plastic. In each cycle, a new PE plastic was colonized, and a new biofilm was formed, allowing for a regular selection cycle of attachment and dispersal. The biofilm populations were serially transferred every 72 h for a total of 40 cycles. Cells that adhered to the tube walls or remained in the planktonic phase were not transferred. Throughout the experimental evolution process, biofilm biomass on the additional PE plastic films, which were independent of the evolutionary transfers, was monitored using OD_600_ measurements of bacterial suspension, taken five times during the 40 cycles (third, 12th, 21st, 31st, 40th). To measure the growth of individual strains during the evolution experiment in carbon-free medium supplemented with PE, bacterial genomic DNA was used. At the end of the experimental evolution, PE plastics with visible biofilms were transferred to disposable culture tubes containing 5 ml of LCFBM with 0.01% yeast extract to promote microbial growth. The culture was vortexed, diluted 1:100 in LCFBM, and 50 μl was plated onto 1:2 diluted lysogeny broth (LB) agar medium for bacterial strain isolation. Single colonies were streaked twice on LB agar media to obtain pure cultures. Overnight cell cultures were then used for Gram staining, following standard protocols.

### Monitor of evolution experiment and strains isolation

For both pure cultures and evolved populations, total genomic DNA was extracted using the OMEGA Soil DNA Kit (M5635-02) (Omega, USA), following the manufacturer’s instructions, and stored at −20°C prior to further analysis. The 16S rRNA genes were amplified using primers 27F and 1492R, following standard protocols. Sanger sequencing of the purified polymerase chain reaction (PCR) products was performed at Sangon Biotech China and further sequences were blasted against NCBI database for strain characterization. Quantitative PCR (qPCR) was performed using species-specific primers targeting the 16S rRNA gene (Supplementary Table 1). Cell growth was quantified for each sample based on 16S rRNA gene copy number per milliliter. All experiments were performed in three biological replicates.

Evolved biofilms were transferred to 12-mm glass coverslips. Bacterial viability within the biofilms was assessed using the Live/Dead BacLight Bacterial Viability Kit (Thermo Fisher Scientific, Waltham, MA, USA), which contains the green-fluorescent nucleic acid stain SYTO 9 and the red fluorescent nucleic acid stain propidium iodide. The staining procedures were performed according to the manufacturer’s instructions. For each biofilm, images were acquired at multiple positions with a default z-step size of 1 μm. Unstained and single-stained control slices for each dye were used to monitor and subtract all corresponding background signals. Quantification of live and dead cells was performed using COMSTAT 2.1 and ImageJ (Fiji distribution).

For high-throughput sequencing, 16S rRNA gene V3–V4 region was amplified using 338F (5′-ACTCCTACGGGAGGCAGCA-3′) and 806R (5′-GGACTACHVGGGTWTCTAAT-3′). PCR amplicons were purified using VAHTS DNA Clean Beads (Vazyme, China) and quantified with the Quant-iT PicoGreen dsDNA Assay Kit (Invitrogen, USA). After quantification, amplicons were pooled in equal amounts, and paired-end 2 × 250 bp sequencing was performed using the NovaSeq 6000 (Illumina) SP Reagent Kit (500 cycles) at Majorbio, Shanghai.

### Polyethylene degradation assays and determination of physical and chemical changes

For the PE powder degradation assay and weight loss analysis, 0.5 g of PE powder (Sigma–Aldrich, Cat:427772-250g, average number-average molecular weight (M_n_) ~ 1700, and weight-average molecular weight (M_w_) ~ 4000) was accurately weighed using a fine scale and transferred into a 50 ml centrifuge tube. To sterilize, 20 ml of 70% ethanol was added to the tube, which was then rotated for 30 min at room temperature. The sterilized PE powder was collected by centrifugation at 12 000 × g for 30 min with slow acceleration and deceleration. After removing the supernatant, the PE powder was air-dried overnight under a biosafety hood. All groups were incubated at 30°C for 30 days with shaking at 120 rpm. The washed residual PE powder was randomly sampled for later analysis.

Molecular weight distribution analysis was performed using high-temperature gel permeation chromatography (HT-GPC, Nexera, Shimadzu) with PE plastic powder after one-month treatment. PE plastic films, after one month of treatment as the sole carbon source, were subjected to the following assays to compare the degradation efficiency under different treatments. High performance XPS (X-ray photoelectron spectrometer, M4 Bruker) was carried out to characterize the binding energy of the substrate. Fourier transform infrared spectroscopy (FTIR) was employed to identify the functional group changes over time. A total of 32 scans were captured using smart endurance single bounce with a diamond tip in the 4000–400 cm^−1^ range. Spectra were analyzed using Spectragryph software (version 1.2.16). Water contact angle (WCA) analysis was used for analyzing surface hydrophobicity of bacteria-treated and -untreated PE plastics. The contact angle of the plastic films with water was measured at room temperature with the measuring device (HE-CA200). All data were collected in triplicate and repeated three times at least.

The surfaces of the PE plastics, along with the structure of the attached biofilm and microbial community, were examined using a scanning electron microscope (SEM). Both uncolonized PE samples and bacteria-treated samples collected after 30 days were coated with a 20 nm layer of gold using an SCD 050 sputter coater (Bal-Tec). Further SEM was conducted with prepared samples (Zeiss Sigma 360) and three samples were used for each treatment.

### Characterization of biofilm formation and cross-feeding experiments

The PE films with biofilm were placed in a 50 ml centrifuge tube containing 40 ml of LCFBM and vortexed for 5 min, then bacterial suspension was centrifugated for 10 min at 10 000 rpm. Pelleted cells were resuspended and counted using the series dilution method of plate counting.

Spent medium were used for cross-feeding experiments. The species were grown in 10 ml LCFBM with commercial PE plastic powder at pH 7 for 2 weeks at 30°C. The cultures were centrifuged at 3220 × g for 10 min, and the spent medium was sterilized using a 50 ml Steriflip filter unit (0.22 μm; Millipore Sigma). To verify sterility, 50 μl of the spent medium was spotted onto LB agar medium. The spent medium was used in place of water in the culture medium. A second preculture was prepared for each species, centrifuged, and inoculated at 10^6^ CFU/ml for a one-week incubation. OD_600_ was measured in different spent media, and productivity was defined as the fold gain in growth (area under the curve of OD_600_ over 24 h), with ratios calculated between two means (*n* = 3) from simultaneous growth assays.

### Exopolysaccharide characterization

Exopolysaccharide collection was performed as described previously [29]. Strains were cultivated at 30°C for PE plastic colonization with shaking at 120 rpm. At certain growth stage, bacteria biomass was suspended in 1 ml of 0.9% NaCl buffer and sonicated (5 × 12 pulses of 1 s at 50% amplitude). Bacterial biomass was separated by centrifugation (10 min at 12 000 × g), and the supernatant was collected. Exopolysaccharide content was quantified using the phenol-sulfuric acid method, with a standard curve constructed from diluted glucose solution [30].

### Characterization of potential polyethylene degradation products

Bacterial cultures were centrifuged at 12 000 × g for 10 min at 4°C to pellet the cells. The resulting supernatants were carefully collected and passed through a 0.22 μm pore-size membrane filter (Millipore) to remove residual cells and debris. For metabolite extraction, 500 μl of the cell-free supernatant was mixed with 1.5 ml of cold methanol (pre-cooled to −20°C) at a 1:3 (v/v) ratio to precipitate proteins. The mixture was vortexed for 30 s, incubated at −20°C for 1 h, and centrifuged at 13 000 × g for 15 min at 4°C. The supernatant was transferred to a clean tube and dried under vacuum using a SpeedVac concentrator. The dried extracts were reconstituted in 100 μl of 50% methanol for liquid chromatography/tandem mass spectrometry (LC–MS/MS) analysis.

Metabolite profiling was performed using an ultra-high-performance liquid chromatography system coupled with a high-resolution mass spectrometer (Thermo Scientific). Chromatographic separation was achieved on a reverse-phase C_18_ column (Waters HSS T3, 2.1 × 100 mm, 1.8 μm) maintained at 40°C. The mobile phases consisted of solvent A (0.1% formic acid in water) and solvent B (0.1% formic acid in acetonitrile). The flow rate was set at 0.3 ml/min with an injection volume of 3 μl. The gradient program was as follows: 0–1.0 min, 98% A; 1.0–8.0 min, linear to 65% A; 8.0–14.0 min, linear to 30% A; 14.0–17.0 min, linear to 2% A; 17.0–20.0 min, hold at 2% A; 20.1–25.0 min, re-equilibration at 98% A. The mass spectrometer was operated with a heated electrospray ionization source in both positive and negative ion modes. The spray voltage was +3.5 kV (positive), −2.8 kV (negative), and the capillary temperature was 320°C, respectively. Full MS scans were acquired at 70 000 resolution over the range 70–1000 m/z.

The raw data were first converted to mzXML format using MSConvert in the ProteoWizard software package (v3.0) and subsequently processed with the XCMS package (v3.12) for feature detection, retention time correction, and alignment. MS/MS spectra were searched against the Human Metabolome Database (HMDB), Kyoto Encyclopedia of Genes and Genomes database (KEGG), mzcloud, and an in-house standard library (Shanghai Personal Biotechnology). MetaboAnalystR 4.0 were used for statistical analysis (principal component analysis, t-tests). Enrichment analysis was performed using the mapped KEGG categories, and pathways showing significant variation (*P* < 0.05 after false discovery rate (FDR) correction) were considered differentially enriched.

For GC–MS analysis of fatty acids, samples were mixed with an internal standard and 2 ml of n-hexane to facilitate fatty acid methyl esterification for 30 min. Subsequently, 2 ml of ultrapure water was added, and the mixture was thoroughly vortexed. A 1000 μl aliquot of the upper organic phase was collected, dried under nitrogen, and re-dissolved in n-hexane. The resulting extracts were subjected to GC–MS analysis.

Chromatographic separation was performed using an Agilent DB-23 capillary column (60 m × 250 μm × 0.15 μm). The initial oven temperature was set to 80°C, ramped to 180°C at 20°C/min, held at 220°C for 8 min, then further increased to 280°C at 5°C/min and held for 3 min. Helium was used as the carrier gas at a constant flow rate of 1.0 ml/min. The MSD ChemStation software (Agilent Technologies) was used to extract chromatographic peak areas and retention times. Quantification of medium- and long-chain fatty acids was performed using calibration curves constructed from authentic standards.

### 16S rRNA gene specific fluorescence in situ hybridization and confocal laser scanning microscopy

Biofilms of evolved populations were formed on PE plastics for 2 weeks to ensure bacterial persistence and activity. Once visible bacterial aggregates formed, the PE plastics were dehydrated by placing them on filter paper and soaking in 75% ethanol for 10 min at room temperature. Labeled oligo probes, Cyanine 5 (Cy5) and 6-car-boxyfluorescein (6-FAM), were synthesized (Sangon Biotech, China) and used for the detection of *Pseudomonas* spp. [31] (Cy5-labeled probes: 5′-GAT CCG GAC TAC GAT CGG TTT-3′), Enterobacteriaceae [32] (6-FAM-labeled probes: 5′-TGC TCT CGC GAG GTC GCT TCT CTT-3′), and Enterococcaceae [33] (6-FAM-labeled probes: 5′-GAA AGC GCC TTT CAC TCT TAT GC-3′).

Fluorescence in situ hybridization (FISH) was conducted as previously reported with modifications [34]. Briefly, biofilms on PE films were firstly fixed with 4% paraformaldehyde overnight at 4°C. After rinsing in phosphate buffered saline (PBS), samples were permeabilized with 1 mg/ml lysozyme (Sigma–Aldrich) for 10 min at room temperature, followed by PBS rinses. Samples were then dehydrated in ethanol series (50%, 70%, 100%) for 3 min each. Hybridization was performed at 46°C for 3 h with a buffer containing 0.9 M NaCl, 20 mM Tris/HCl (pH 7.2), 30% formamide, 0.01% Sodium dodecyl sulfate (SDS), and fluorochrome-labeled probes (5 ng/μl). Post-hybridization, PE samples were rinsed in washing buffer (20 mM Tris/HCl, 5 mM EDTA, 102 mM NaCl) at 48°C, incubated for 15 min, and rinsed with ice-cold dH_2_O. FISH samples were imaged with confocal laser scanning microscopy (CLSM) (LSM 800, Zeiss) and recorded Z-stacks for 3D visualization with 1 μm steps. Stacked images were merged and analyzed using ImageJ software as previously reported [29].

### Transcriptomic analyses

The SynCom and the St monocultures were incubated on PE plastics in LCFBM medium for a week, which were exploited as experimental groups. In contrast, populations and single strains grown in LB medium were used as a control group. Briefly, total RNAs were extracted using TRIzol reagent (Invitrogen, USA) and DNA contamination was removed using MEGAclear Kit (Life technologies, USA). RNA concentration and purity were measured with a NanoDrop 2000 (Thermo Scientific, USA), and integrity was assessed by Agilent 2100 Bioanalyzer using the RNA 6000 Nano Kit (Agilent Technologies, USA). Ribosome RNA depletion was performed using the RiboCop rRNA Depletion Kit (Lexogen, USA), followed by fragmentation of mRNA into 200 nucleotide fragments. Double-stranded cDNA was synthesized with random hexamer primers (Illumina), incorporating dUTP in the second strand. RNA-seq libraries were prepared using the Illumina Stranded mRNA Prep kit and sequenced on a NovaSeq 6000 (Illumina). Low-quality reads, reads with >10% N bases, and adaptor sequences were removed from the data. For St monocultures, high quality reads in each sample were mapped to the reference genome (GCF_019702405.1) using Bowtie2. Gene and transcript abundances from RNA-Seq data are quantified using RSEM, which employs the expectation maximization algorithm to estimate abundances, accounting for paired-end reads, fragment lengths, and quality scores. Expression levels are calculated using fragments per kilobase per million mapped reads and transcripts per million, which normalize for gene length and sequencing differences, allowing for direct comparison of gene expression across samples. Differentially expressed genes (DEGs) were identified using the edgeR, DESeq2, or DESeq packages for each dataset and alignment/quantification protocol. As for functional characterization of DEGs, KOBAS, and Goatools were used to identify significantly enriched KEGG functions and GO terms, respectively.

## Results

### Parallel evolution of bacterial populations on polyethylene plastics

**Figure 1:**
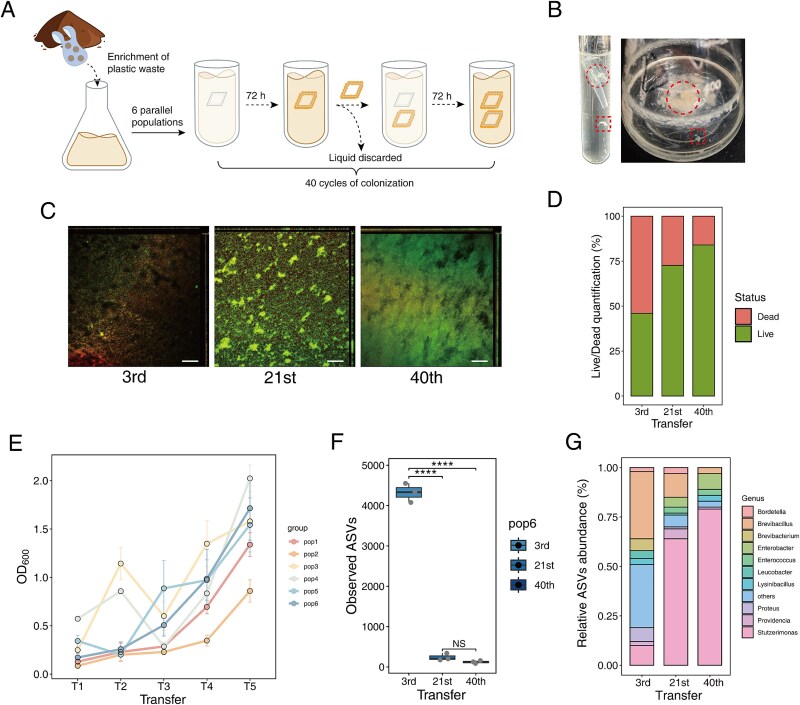
An analysis of the evolutionary process shapes biofilms on PE plastic surfaces. (A) Description of the specific process of experimental evolution, associating with bacterial biofilm formation and dispersal of cells. (B) After experimental evolution, dense biofilms (dashed line circles) and dispersed bacterial aggregates (dashed line squares) formed on PE plastic surfaces. (C) CLSM of live/dead biofilm cells at three time points during the evolution experiment. Live (green) and dead (red/yellow) bacterial cells were visualized. Scale bars: 20 μm. (D) COMSTAT analysis of biofilm biomass. The percentage of live and dead cells was calculated from each of six z-stacks obtained from three independent biological replicates. (E) Quantitative analysis of biofilms on PE plastic surfaces at five stages during evolution. (F) 16S rRNA gene amplicon sequence analysis of population 6 indicated that bacterial alpha diversity within the biofilm rapidly decreased as experimental evolution progressed. (G) Analysis of bacterial community composition at the genus level in biofilms as the experiment progressed. Error bars represent standard error of the mean of three biological replicates (*n* = 3). ^****^P < .0001, NS: not significant, based on ANOVA analysis.

To identify bacterial communities capable of forming biofilms and degrading PE, we collected plastic debris from contaminated sediment in Dianchi Lake, Yunnan, China. After an enrichment experiment with PE plastics, we established six parallel bacterial cultures and subjected them to experimental evolution under controlled laboratory conditions. Strains that successfully colonized PE formed multi-species biofilms within 72 h. These biofilms were transferred to fresh tubes with new medium and PE for re-colonization (Fig. 1A). After the experimental evolution process, the evolved microbial communities formed significantly enhanced biofilms on PE surfaces in test tubes and conical flasks (Fig. 1B). The formation of bacterial aggregates further demonstrates a shift in the microbial community towards a multicellular lifestyle (Fig. 1B). To monitor the biofilm development during the evolutionary process, both qualitative and quantitative analyses were conducted using Live/Dead staining combined with CLSM. The thickness of the biofilm gradually increased with evolutionary time (Fig. 1C). As key strains became dominant, the biofilm at the 21st cycle exhibited the formation of cell aggregates, whereas the biofilm at the 40th cycle reached the maximum thickness. Quantitative analysis further revealed that, although the initial biofilm contained a substantial proportion of dead cells, the proportion of live cells increased progressively throughout the evolutionary selection cycles (Fig. 1D). All six evolved populations increased biofilm productivity in the experimental evolution regime, suggesting improved PE degradation and possible synergy among the strains (Fig. 1E).

To study the evolutionary dynamics of bacterial communities, biofilms were collected at three time points from population 6, chosen for its consistently increasing biofilm production. Total DNA was then extracted for 16S rRNA gene amplicon sequencing. Alpha diversity indices were used to analyze changes in biofilm complexity. Compared to the ancestral populations, the evolved populations showed a sharp decline in community richness and diversity between third and 21st Transfer, with no significant changes between stages 21st and 40th Transfer for observed amplicon sequence variants (ASVs) (Fig. 1F) and Shannon index (Supplementary Fig. 1A). Beta diversity analysis also revealed significant separation in the early stages of evolution (Supplementary Fig. 1B). These results suggested that the experimental conditions created a strong evolutionary bottleneck, driving rapid adaptation to the environment, where hydrocarbons were the sole carbon source. Furthermore, *Stutzerimonas* species became dominant during experimental evolution, comprising over 60% of the community by the mid-phase, indicating their fitness advantage (Fig. 1G).

**Figure 2:**
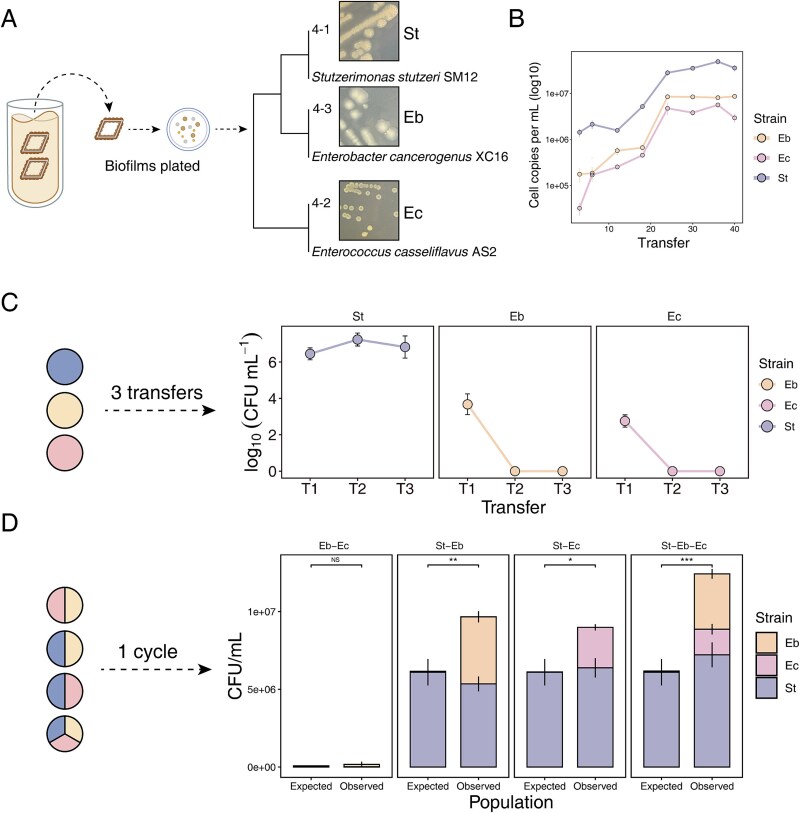
Three dominant bacterial strains were isolated from the group with the highest biofilm production, and their stability was analyzed. (A) The biofilm on the final piece of plastic was vigorously vortexed, plated on LB agar, and the bacteria were isolated and identified. Identification of isolated strains based on Sanger sequencing of the 16S rRNA gene followed by sequence alignment against the NCBI database and phylogenetic tree construction using the maximum likelihood method. (B) Cell growth of each species in the experimental evolution system of biofilm population 6, as indicated by the 16S rRNA gene copy number. (C) Stability of biofilm formation by isolated strains using PE as the sole carbon source (transferred three times). (D) The expected productivity was calculated by multiplying the initial proportion of each morphotype in the founding population by its monoculture yield (CFU per milliliter). ^*^P < .05, ^**^P < .01, ^***^P < .001, ns: not significant, based on *t*-test.

At the end of the experiment, bacterial biofilms on the PE surface were collected in PBS buffer and vortexed with glass beads to detach the cells from the PE substrate. The resulting bacterial suspension was streaked onto LB agar plates to obtain culturable bacterial isolates. Through 16S rRNA gene amplification and sequencing, three isolates were identified: *Stutzerimonas stutzeri* (St), *Enterobacter cancerogenus* (Eb), and *Enterococcus casseliflavus* (Ec). After 48 h of incubation, St colonies appeared pale yellow, 4–6 mm in size with rugose colony morphology. Eb colonies were larger, semi-transparent with clear edges, whereas Ec colonies were the smallest, about 2 mm in diameter, yellow in color, and smooth (Fig. 2A). We also tracked the dominant species over 40 serial transfers using 16S rRNA gene-based qPCR. As expected, the cell numbers of all three species increased significantly after 40 transfers compared to the initial stage (Fig. 2B). Furthermore, all three species exhibited rapid growth by the 20th transfer and remained stable thereafter.

To test the PE colonization and degrading ability of the isolated strains, monocultures were grown with PE plastic as the sole carbon source, allowing biofilm formation. Biofilms were transferred three times following the experimental evolution protocol, and bacterial counts were measured as colony-forming units (CFU) per milliliter. Only St consistently utilized PE and formed stable biofilms across all three transfers. In contrast, Eb and Ec formed biofilms during the initial inoculation but showed insufficient biofilm formation after two subsequent transfers on new plastic surfaces (Fig. 2C). This inability to persist may be due to their limited capacity to use PE as a carbon source, especially during the re-colonization stages.

To explore potential interactions among these isolates on PE surface, we tested pairwise co-cultures and the three members synthetic community (SynCom) for their ability to utilize PE and form biofilms. Although the co-culture of Eb and Ec led to a slight increase in biofilm production, the difference was not significant compared with the expected yield (Fig. 2D). However, when St was present, all co-culture combinations showed a significant increase in biofilm production compared with the expected yield based on monoculture productivity (Fig. 2D). This indicated that St plays a dominant role in promoting biofilm growth, leading to a more than 2-fold increase in the total population size, facilitated by potential complementarity. St appears to act as a keystone species as this species increased the growth of the other two species, thereby promoting synergy in biofilm formation. We hypothesized that St is primarily responsible for PE degradation, whereas Eb and Ec cannot grow on PE when cultured individually but may benefit from utilizing metabolites released by St during co-cultivation.

### Evolved bacterial consortia promoted polyethylene plastic degradation

Whereas PE plastic serves both as an attachment surface and as the sole carbon source, increased biomass likely reflects enhanced degradation. St plays a key role in the microbial community due to its consistent colonization of PE. Therefore, we compared the degradation efficiency of St monoculture with the evolved microbial community.

SEM images showed depressions on the PE surface treated with St, in contrast to the smooth surface of the control, suggesting potential surface alteration by St (Supplementary Fig. 2A). SynCom created more extensive surface alterations on PE, suggesting a greater degree of surface modification compared to the individual strain. Hydrophobicity, measured by WCA, decreased from 105.1 ± 1.4 in the control to 101.1 ± 1.0 in St-treated and to 96.1 ± 2.2 in SynCom-treated samples, with the latter showing a greater reduction (Supplementary Fig. 2B). The samples treated with microbial community showed a greater reduction in hydrophobicity compared to St alone.

**Figure 3:**
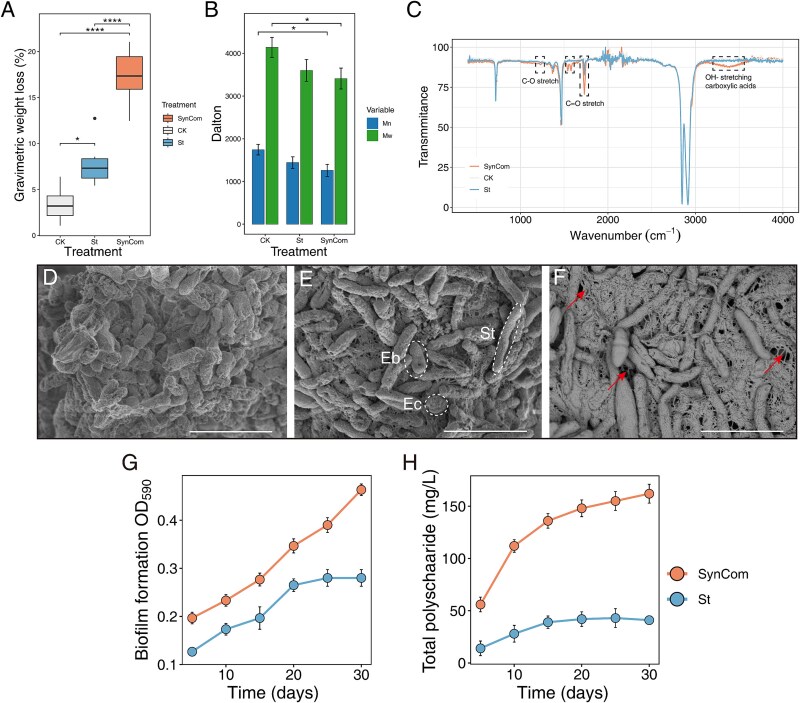
PE degradation and biofilm formation dynamics of St and SynCom on the PE surface. (A) Gravimetric weight loss of PE plastic treated with microbial biofilms. (B) Changes in the molecular weight of standard PE in different treatment groups after one month, as determined by GPC analysis. (C) FTIR spectrum showed PE functional group changes in the bacteria treated PE samples. (D−F) St, SynCom, and extracellular matrix imaging in SEM, respectively. (G, H) Time-series analysis of biomass and total extracellular polysaccharides, respectively. ^*^P < .05, ^****^P < .0001, based on ANOVA analysis.

Weight loss experiments using PE powder also confirmed higher PE degradation by the community than by St alone (Fig. 3A). HT-GPC revealed that both the Mw and the Mn of PE powder were significantly reduced after biological treatment compared to the untreated group (Fig. 3B). Among the treatments, SynCom-treated PE showed a significantly greater reduction in both parameters compared to the control (CK), as determined by an unpaired t-test. These results indicate that the PE powder underwent substantial depolymerization under microbial treatment. FTIR was applied to analyze changes in the surface chemical composition and functional groups of PE plastic (Fig. 3C). In the St-treated samples, the FTIR spectra revealed two distinct peaks: one at 1650 cm^−1^ corresponding to C=O stretching, and another at 1120 cm^−1^ corresponding to C−O stretching. Additionally, in the SynCom-treated samples, a broad peak appeared in the 3000–3600 cm^−1^ range, corresponding to OH− stretching from carboxylic acids. This suggested that the microbial community-treated PE underwent a higher degree of oxidation and more extensive degradation compared to the St-treated samples.

### Synergistic biodegradation was achieved by promoted exopolysaccharide secretion of evolved consortium

Both St and the SynCom formed microcolonies aiding in plastic degradation (Supplementary Fig. 3A and B), but at higher magnification, structural differences were apparent. St degraded plastic without a significant EPS network (Fig. 3D). In contrast, the SynCom formed a dense biofilm composed of the three strains, including elongated St cells, connected by EPS, improving coverage and degradation efficiency (Fig. 3E and F). St likely directed its metabolic resources primarily towards producing degradation-related enzymes rather than extracellular matrix.

We then compared the biofilm growth and total polysaccharide production, which reflected EPS production, during colonization. By day five, both St and the SynCom showed sufficient bacterial attachment to the PE plastic (Fig. 3G). However, the SynCom consistently maintained a higher bacterial abundance, whereas St reached a stable phase around Day 20 with no further growth, the SynCom continued to increase steadily (Fig. 3G). Additionally, The SynCom produced more total carbohydrate on PE plastic. Over the 30-day period, St’ carbohydrate production increased gradually from 14 mg/L to about 40 mg/L, whereas carbohydrate production of the SynCom peaked at around 162 mg/L (Fig. 3H). This significant difference aligned with the SEM observations, indicating that the metabolic cooperation within the SynCom enhanced EPS secretion and biofilm formation. The multispecies biofilm structure may enhance mass transfer by reducing diffusion path lengths and establishing localized microenvironments that support enzymatic reactions, which are often rate-limiting steps in plastic degradation.

### Surface chemical transformations and metabolic signatures of biodegraded polyethylene

**Figure 4:**
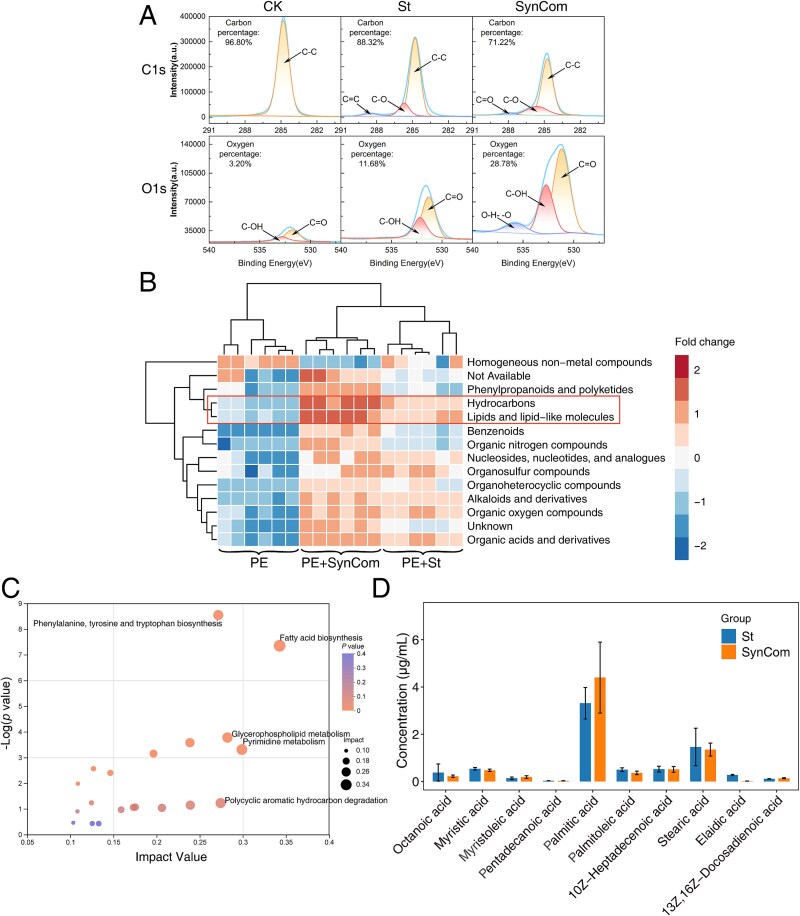
Oxidation levels and intermediate product identification during the one-month degradation of PE powder. (A) XPS spectra of untreated control PE powder (CK) and PE powder degraded by St and SynCom. Binding energy ranges are specified, with peak-fitted spectra of carbon and oxygen. (B) Extracellular non-targeted metabolomics analysis by LC–MS of degradation products. A heatmap of metabolites based on the HMDB database shows clustering of untreated, St-treated, and SynCom-treated groups. (C) KEGG pathway topology analysis of differential metabolites between the two biologically treated groups and the untreated control. (D) Absolute quantification of medium- and long-chain fatty acids in the two biologically treated groups based on extracellular targeted metabolomics using GC–MS.

The XPS analysis of medium-treated (CK) and biologically treated PE films highlighted marked chemical modifications consistent with oxidative chain scission and functionalization (Fig. 4A). In the O 1s region, the spectrum of biologically degraded PE could be deconvoluted into two dominant peaks at 531.2 eV and 532.8 eV, attributable to C=O (carbonyl) and C–OH (hydroxyl) bonds, respectively. Both signals were significantly enhanced in the St and SynCom treatments compared to CK, reflecting an increased surface oxygen content and the formation of new oxygenated moieties. Correspondingly, in the C 1s region, the degraded samples exhibited a reduction in the C–C/C–H peak at 284.8 eV alongside the emergence and growth of C–O (285.9 eV) and a minor C=O (ketone, 287.2 eV) peak, indicating oxidative depolymerization and insertion of polar functional groups. These spectral shifts collectively demonstrate that microbial colonization promotes surface oxidation and chain-end attack mechanisms during PE biodegradation.

To uncover potential intermediate degradation products, we employed LC–MS/MS to profile extracellular metabolites in the culture supernatant. Both St and SynCom treatments yielded a diverse spectrum of small molecules, with pronounced enrichment of hydrocarbons, mono and polyunsaturated fatty acids, and other lipid-like compounds relative to control (Fig. 4B). KEGG pathway enrichment analysis revealed that the enriched metabolites were significantly associated with polycyclic aromatic hydrocarbon breakdown and fatty acid biosynthesis pathways (Fig. 4C), underscoring the link between surface oxidation of PE chains and subsequent microbial uptake and transformation of oxygenated fragments into central metabolic intermediates.

For absolute quantification of candidate medium- and long-chain fatty acids, GC–MS/MS was performed using calibration curves constructed from 45 authentic standards. Ten metabolic intermediates were detected above the quantification limit, with palmitic acid (C_16_H_32_O_2_) exhibiting the highest concentrations with 3.31 μg/ml in the St treatment and 4.39 μg/ml under SynCom (Fig. 4D). Stearic acid (C_18_H_36_O_2_) similarly accumulated at elevated levels in both biologically treated groups. Aside from octanoic acid (C_8_H_16_O_2_), all identified intermediates ranged from C_14_ to C_18_, consistent with terminal and subterminal oxidation of PE polymer chains. The detection of these oxidized carboxylic acids provides compelling evidence that microbial PE degradation proceeds primarily via exo-type oxidative attacks at polymer chain ends, generating soluble medium- and long-chain fatty acid intermediates that are further assimilated and catabolized by bacteria.

### Evolved community synergy is attributed to cross-feeding and spatial partitioning

**Figure 5:**
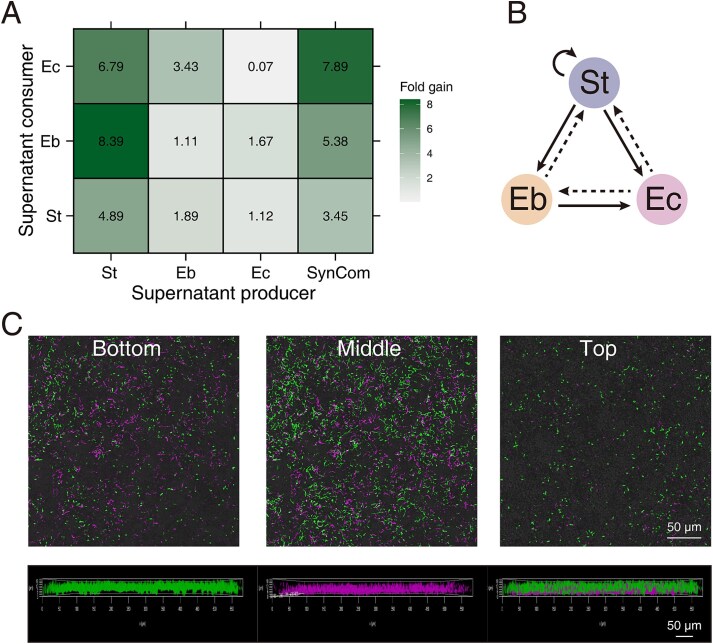
Cross-feeding interactions between isolates and CLSM imaging analysis of biofilm structure. (A) Pair-wise culturing assay with the numerical values represents the fold increase in growth in the supernatant relative to the growth in unconditioned medium. (B) Bacterial interaction diagram: solid lines indicate promotion, whereas dashed lines represent neutral effects (no promotion). (C) FISH–CLSM imaging of SynCom with St false colored in magenta, whereas the other two strains in green.

The increased biofilm production and corresponding enhanced degradation efficiency might be the result of two key factors: more efficient nutrient utilization and better use of limited space. To quantify the effects of cross-feeding alone, we measured the growth of St, Eb, and Ec in the spent medium supernatants from both their own and the other isolates. All isolates—except Ec in its own spent medium—showed enhanced growth in supernatants compared to the original PE medium (Fig. 5A). However, the degree of growth enhancement varied, with benefits being asymmetrical. St grew best in its own supernatant, increasing its productivity nearly 5-fold compared to unconditioned medium, which is expected given its primary role in degrading PE and dominating the evolved population. In contrast, Eb grew optimally in St supernatant, whereas Ec thrived in the spent medium of the SynCom. Ec benefited significantly from the metabolic by-products of St and Eb, forming an interdependent food web in which only St can sustain itself independently (Fig. 5B).

To investigate how the isolates partition biofilm space, we used FISH combined with CLSM to image the mixed population. Whereas St and the other two isolates exhibited distinct growth patterns during biofilm assembly, St was stained with the Cy5(magenta), whereas Eb and Ec were stained with 6-FAM (green). St and the other two isolates contributed to the biofilm structure in distinct ways (Fig. 5C). St adhered more effectively to the plastic surface, spreading horizontally rather than growing vertically. In contrast, Eb and Ec formed the tallest and densest aggregates, covering the biofilm surface. These findings suggested that St occupied the basal layer of the biofilm, degrading the PE plastic, whereas Eb and Ec attached to the degradation by-products and formed higher aggregates that encapsulated St and the plastic. This spatial structure may create a favorable microenvironment for biofilm-mediated PE degradation, potentially enhancing local enzyme accumulation within the biofilm. After disrupting the SynCom structure (biofilms were sonicated and transferred), total biofilm production gradually decreased, eventually leading to biofilm collapse (Supplementary Fig. 4).

### Gene expression of St vs. SynCom during polyethylene plastics degradation

**Figure 6:**
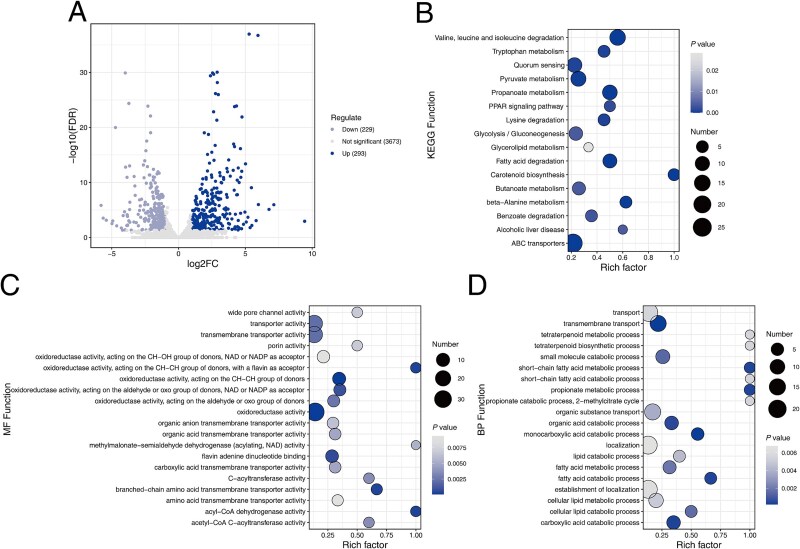
Volcano plots and heatmaps of DEGs and functional gene enrichment analysis. (A) Volcano plot of DEGs in St during PE plastic degradation compared with growth in LB medium. (B) Enriched analysis of KEGG functions in St during PE plastic degradation compared with growth in LB medium. (C) Enriched molecular functions of GO terms in St during PE plastic degradation compared with growth in LB medium. (D) Enriched biological process of GO terms in St during PE plastic degradation compared to growth in LB medium.

Differential gene expression was recorded in St alone and the SynCom, and calculated as log2 fold change. Principal component analysis using the Bray–Curtis distance matrix was performed to assess expression profile variation. Differential gene expression analysis using DESeq2 identified 293 genes that exhibited significantly increased transcript levels under PE colonizing conditions compared to the reference nutrient-rich medium (*P* < .05, Dataset S1). The transcriptional fold changes of these genes ranged from 2 to 141. In contrast, transcription of 229 genes were downregulated, and 3673 genes showed no significant transcriptional difference between the two growth conditions (Fig. 6A). Genes differentially expressed in St cells cultured under PE-colonizing and planktonic lysogenic broth (LB) conditions exhibited two distinct patterns (Supplementary Fig. 5).

Following annotation, KEGG and gene ontology (GO) enrichment analyses were conducted to identify functional changes in cells grown in the presence of PE. Genes with higher expression levels mapped to 16 significantly enriched KEGG pathways. These included pathways related to PE degradation, such as fatty acid metabolism and the breakdown of PE by-products (pyruvate, propanoate, and butanoate). In addition to pathways associated with bacterial activities, such as quorum sensing and macromolecular transport (e.g. ABC transporters) (Fig. 6B). The GO enrichment analysis identified 80 enriched GO terms (Dataset S2). The top 20 enriched molecular functions included transport of by-products, including wide pore channels, transmembrane transporters, and organic and amino acid transport (Fig. 6C). Most remaining molecular function pathways related to the oxidation of PE, targeting CH–OH or CH–CH groups, as well as beta-oxidation of PE by-products. Biological processes were strongly enriched in the metabolism of fatty acids, organic acids, monocarboxylic acids, lipids, and carboxylic acids, all involved in the depolymerization and cellular metabolism of PE (Fig. 6D). The most enriched biological process pathways were related to short-chain fatty acids, propionates, and tetraterpenoids. The *prp* operon, consisting of five genes (*prpB*, *prpC*, *acnD*, *prpF*, and *prpD*) in *Pseudomonas* spp., is responsible for propionate metabolism, which is a downstream product of odd-chain-length alkane oxidation. All five genes exhibited significantly increased transcript abundance in the presence of PE, suggesting a core degradation cycle and energy route producing pyruvate (Supplementary Fig. 6).

**Figure 7:**
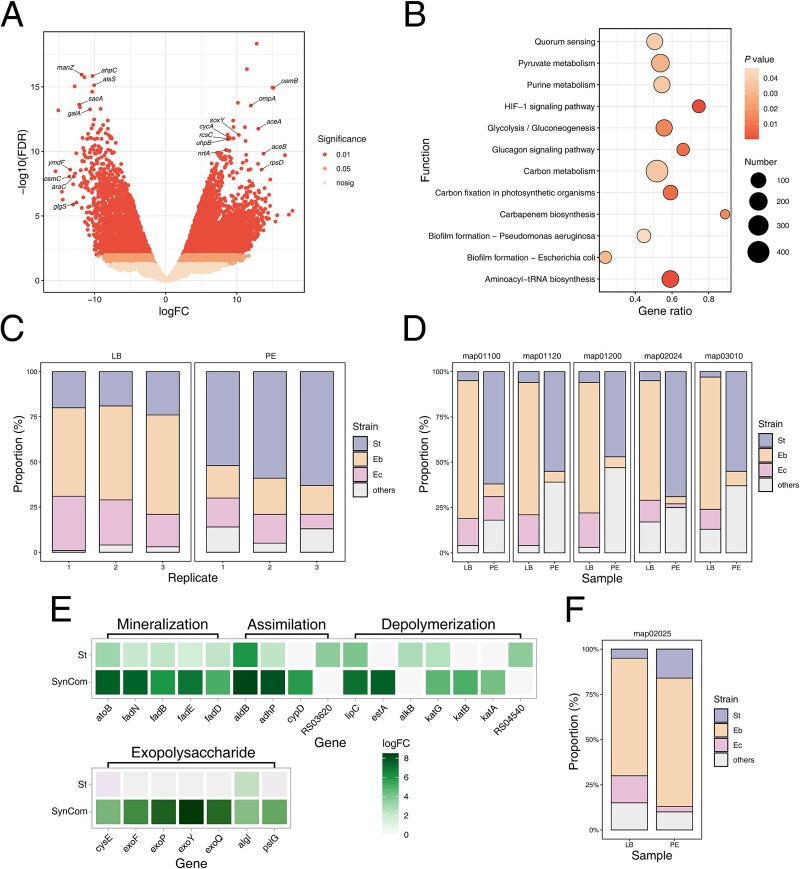
Functional and expressive contribution analysis of metatranscriptomic data. (A) Volcano plot of DEGs in the SynCom during PE plastic degradation compared to growth in LB medium. (B) Enriched analysis of KEGG functions in SynCom during PE plastic degradation compared to growth in LB medium. (C) The contribution proportions of three isolates to the total transcripts in the SynCom during PE degradation and LB culture. (D) The contribution proportions of three isolates to the total transcripts in the top five KEGG pathways under two culture conditions. KEGG pathway annotations include: map01100 (metabolic pathways), map01120 (microbial metabolism in diverse environments), map01200 (carbon metabolism), map02024 (Quorum sensing), and map03010 (ribosome). (E) Differential expression of genes related to PE degradation and exopolysaccharides under PE degradation conditions compared to LB conditions. (F) Relative KEGG pathways of isolates expressive contributions. map02025 (biofilm formation—*Pseudomonas aeruginosa*).

We tested the expression patterns of the SynCom community and the function-species correlations during PE utilization. After quality control and assembly with Trinity, differential expression analysis (edgeR, FDR < 0.05, |log2FC| > 2) identified 30485 transcripts, including 9660 increased transcript abundance and 4518 decreased (Fig. 7A). Higher expressed genes, categorized via KEGG, were predominantly associated with quorum sensing, biofilm formation, and carbon metabolism (Fig. 7B). GO analysis revealed enriched functions in PE-treated samples, particularly in transferase activities and membrane-related processes, underscoring the role of intercellular communication in PE degradation (Supplementary Fig. 7). Species-specific expression patterns showed dominant genes from Eb in LB and St in PE (Fig. 7C and D), highlighting *Stutzerimonas*’ contributions to key metabolic pathways in the presence of PE were adaptive.

To compare the degradation processes between monocultures and the bacterial community, gene expression profiles involved in specific degradation pathways were analyzed. During the depolymerization of PE, the *katABG*, *estA*, and *lipC* genes had increased transcript abundance in the SynCom, whereas *alkB* showed increased transcript abundance in St monocultures (Fig. 7E). SynCom and St appeared to rely on different extracellular enzymes to introduce oxygen into the hydrocarbon chain and depolymerize PE into low-molecular-weight polymers. In the subsequent oxidation or assimilation process, different enzymes are involved, with P450 hydroxylase (*cypD*) and monooxygenase (RS03620) having significantly increased transcript abundance in SynCom and St, respectively. In the following reaction chain, particularly in the β-oxidation process, the genes involved had significantly increased transcript abundance in both the SynCom and St. Additionally, given the significant secretion of extracellular matrix in the SynCom compared with the St monoculture, expression of genes related to exopolysaccharide production was also examined. In St, only the gene associated with alginate transferase had significantly increased transcript abundance, whereas in the SynCom, seven genes related to exopolysaccharide production had increased transcript abundance, confirming the results related to increased biofilm matrix production in the SynCom (Fig. 7E). In the KEGG function (map02025) related to biofilm formation, Eb played a dominant role rather than St, suggesting a task division within the SynCom (Fig. 7F).

**Figure 8:**
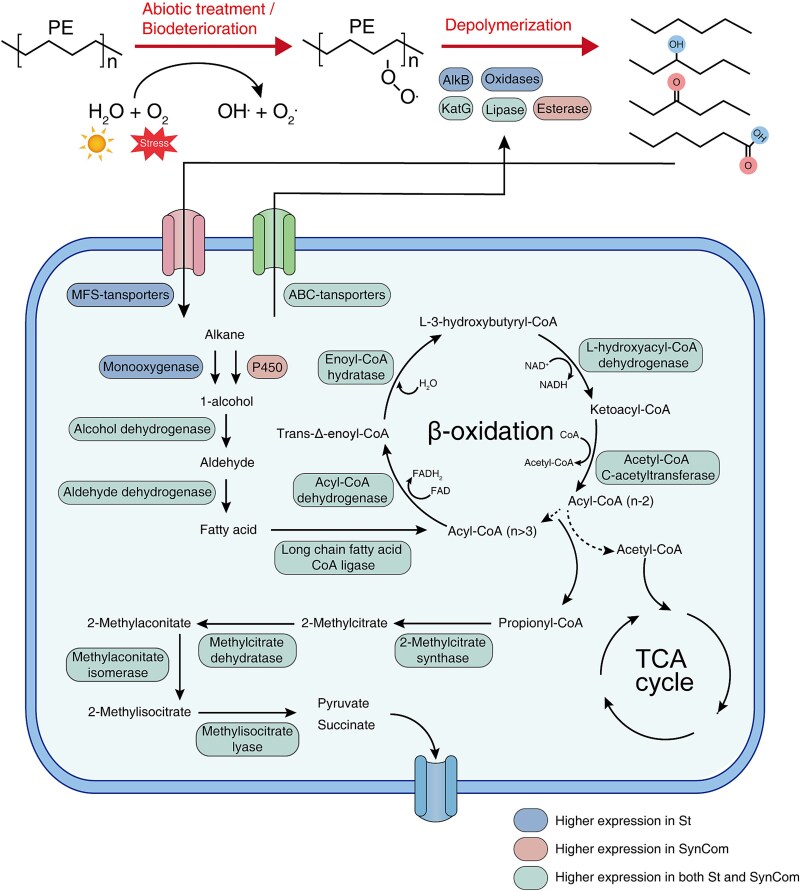
Conceptual model of St and the SynCom utilizing PE as the sole carbon source during PE degradation. The blue, red, and green boxes represent features identified only in St, only in the SynCom, and in both St and the SynCom, respectively.

Based on our experimental results, a conceptual model and central metabolic pathways for PE degradation by St and the SynCom can be proposed (Fig. 8). The most significant difference between the degradation processes of monocultures and the bacterial community is the involvement of extracellular enzymes, including catalase-peroxidase KatG, monooxygenases, lipases, and esterases. After the absorption of small-molecule alkanes, St and the SynCom relied on monooxygenases and cytochrome P450 enzymes, respectively, to produce alcohol. In the later stages of metabolism, key genes had significantly increased transcript abundance in both St and the SynCom.

## Discussion

Bacterial metabolic cooperation in biofilms has been described in various natural and artificial environments, including food [35, 36], plant roots [37], and laboratory systems [38, 39]. However, aside from bioinformatics models [40], few studies have employed experimental evolution as a screening strategy to develop biofilm-based synthetic consortia for plastic degradation. The primary finding of our study is that experimental evolution effectively creates metabolically cooperative bacterial populations. The enrichment of bacterial plastic-degrading trait is associated with environmental plastic pollution, highlighting that microorganisms promptly adapt to these selection pressures over time [41]. As a result, several studies have used plastic biofilms to isolate plastic-degrading bacteria successfully [42, 43]. One key benefit of microbial communities, compared to single strains, is that microbes often exchange metabolic byproducts, which leads to cross-feeding [19, 44]. For example, in nitrogen-poor environments, nitrogen-fixing bacteria and degraders can work together to break down pollutants [45]. In marine microbial communities, cross-feeding of metabolites like n-alkanals and aromatics aids bacterioplankton formation [46]. Studies on cross-feeding showed that different species specialize in certain metabolic pathways, like amino acid or sugar synthesis, making them more efficient [47–49]. Our results indicate that the constructed SynCom exhibits functional division of labor, which facilitates its colonization and degradation of the PE surface. Metabolic cooperation benefits the community composed of *Stutzerimonas*, *Enterobacter*, and *Enterococcus* species (Figs. 2 and 3). Specifically, *Stutzerimonas* and its byproducts in the supernatant help the whole community to grow and therefore act as a keystone species. Microbial communities often rely on a few specialized primary degraders to break down complex substrates into small metabolites such as monosaccharides or short-chain fatty acids [50]. These metabolites are shared through cross-feeding, providing energy and nutrients to the wider community and thereby sustaining its stability and function [51].

Moreover, repeated selection through experimental evolution led to a stable formation of structured communities on plastic surfaces, underscoring the importance of structured population formation for the persistence and functionality of enriched communities [52]. CLSM imaging revealed distinct spatial distribution of St, Eb, and Ec within the biofilm, suggesting an ecological task division similar to differentiating ecotypes of experimentally evolving biofilms [26, 27, 53]. St initiates the degradation of PE plastic and is predominantly localized at the base of the biofilm, near the plastic substrate. In contrast, Eb and Ec form the protective outer layers that potentially enhance the biofilm's stability and thickness. Moreover, both biomass and extracellular matrix production are significantly higher in the SynCom compared with the St monoculture. The biofilm matrix is generally considered as a shared resource that supports biofilm structure and enhances extracellular enzyme activity by facilitating mass transfer [54]. Moreover, increased biofilms could potentially concentrate the PET degrading enzymes locally around the target substrate and retain it within the biofilm matrix for extended periods, thereby enhancing the overall rate of plastic degradation [55]. Such cooperative interaction not only maximizes the biofilm’s structural resilience but also creates a microenvironment that enhances the degradation process, which could be leveraged in future bioremediation strategies. Similarly, a previous study reported that biofilm culture methods are more effective than enrichment methods for studying community-level interactions in polycyclic aromatic hydrocarbon (PAH)-degrading microbial communities, as biofilm culture better preserved active bacterial and genetic diversity [56]. This is attributed to a major concern of enrichment assays: the emergence of so called “cheaters” that exploit benefits produced by the “cooperators.” In well-mixed, unstructured environments cooperators are more susceptible to exploitation by cheaters [57]. In the described SynCom, *Stutzerimonas* acts as a public producer of PE degrading enzymes, whereas Eb and Ec can be considered as either commensals or exploiters. Our results further demonstrated that when the biofilm structure is disrupted, the cooperative species diminishes, potentially leading to biofilm collapse. Therefore, establishing a structured microbial community that maximizes cooperation is essential to ensure efficient PE degradation as demonstrated in our work.

We dissected the molecular mechanisms through analyzing the gene expression networks of St and SynCom under PE induction. Although previous studies have demonstrated the PE-degrading capability of *Stutzerimonas* spp. [58, 59], our study reveals the key pathways and specific genes involved in *Stutzerimonas*’ degradation capability of PE. St had increased expression of genes related to pathways for alkane degradation and β-oxidation, particularly in the metabolism of even- and odd-numbered carbon fatty acids. Similar to a previous study [60], the upregulation of the *prp* operon underscored the importance of odd-numbered fatty acid metabolism in PE plastic degradation that indicates a complete degradation of saturated fatty acids.

Metatranscriptomics facilitates the study of multispecies biofilm communities, particularly in laboratory settings [61]. In our study, biofilm-associated genes, including those involved in quorum sensing, were notably enriched during the degradation process. Nonetheless, whether biofilm-associated genes mainly support surface attachment or also influence degradation-related gene expression remains unclear and requires further controlled experiments. Analysis of the transcript-to-species ratio indicated that Eb transcription dominated in LB medium, whereas St dominated during PE degradation, suggesting that metabolic cooperation is evolutionarily shaped and specifically observable on the PE surface. Differences in Carbon metabolism and Microbial metabolism in diverse environments indicate that the functional distribution of the SynCom during PE degradation diverges from that observed in LB. Quorum sensing (map02024) also showed functional shifts in the strains across the used cultivation media. LB disrupted both community structure and nutrient composition, which are the two most critical factors shaping community cooperation [62]. Comparison of specific genes involved in the degradation process between the SynCom and St revealed that the upregulation of genes related to PE degradation was higher in the SynCom than in the St monocultures, correlating with higher biomass of the SynCom. Regarding the key gene pathways for PE degradation, St and the SynCom showed identical pathways after assimilation and mineralization, consistent with previous reports [60, 63]. Nevertheless, we observed differential expression of key enzymes, including oxidases, esterases, cytochrome P450, and monooxygenases, during the polymerization process in monocultures and microbial communities, suggesting that the presence of other species influenced the enzyme secretion of St. Understanding why these shifts occur will require further analysis, incorporating additional data such as metaproteomics. Through analyzing exopolysaccharide gene and transcript distribution, we found that exopolysaccharide production is primarily enriched in Eb rather than St, providing molecular evidence of task division within the SynCom. Although the link between bacterial members and functions has been explored, identifying strain-specific gene expression is still limited by technical challenges. Genome-resolved approaches with genomic markers could improve resolution and should be further investigated.

In summary, we experimentally evolved natural biofilm populations on PE plastics to describe the molecular mechanisms of community functioning during PE degradation, which has important implications for building artificial microbial communities. The metabolic cooperation within these communities, leading to functional specialization, improves biofilm formation and plays a key role in refining conditions for optimal plastic degradation. In addition, multiple transcripts encoding extracellular proteins showed PE-specific responses, potentially indicating a transcriptional basis to produce a mixture of enzymes involved in PE degradation.

## Supplementary Material

Supplementary_file_wraf223

Dataset_S1_wraf223

Dataset_S2_wraf223

## Data Availability

The 16S rRNA gene sequence data have been deposited in the NCBI database under accession number: PRJNA1154807. The transcriptomics data of monocultures and cocultures have been deposited in the NCBI database under accession number: PRJNA1153981 and PRJNA1154404.
