# Supplementary material for: Synergistic biodegradation of polyethylene by experimentally evolved bacterial biofilms
Source: ISME J. 2025 Oct 8;19(1):wraf223. doi: 10.1093/ismejo/wraf223 (PMC12596105; doi:10.1093/ismejo/wraf223)
Supplement: Supplementary_file_wraf223 [file supplementary_file_wraf223.pdf]

Supplementary figures

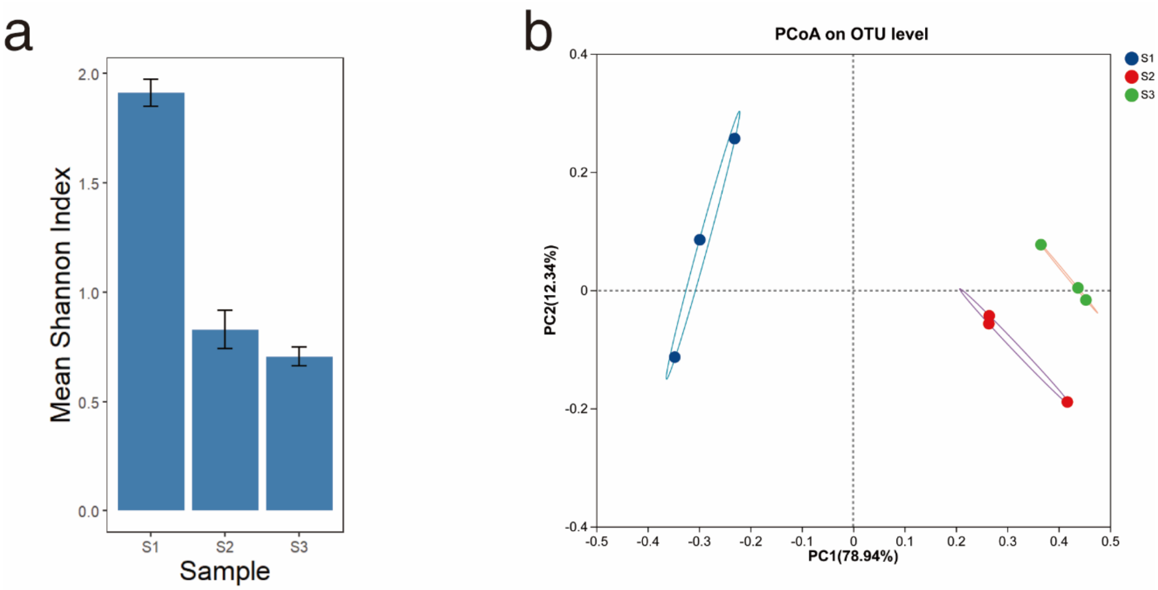

Figure S1. Analysis of microbial community diversity of population 6 during evolution. Three replicates were taken for each time points. (a) Shannon indices at three time points during evolution. (b) PCoA analysis of the evolved microbial community.

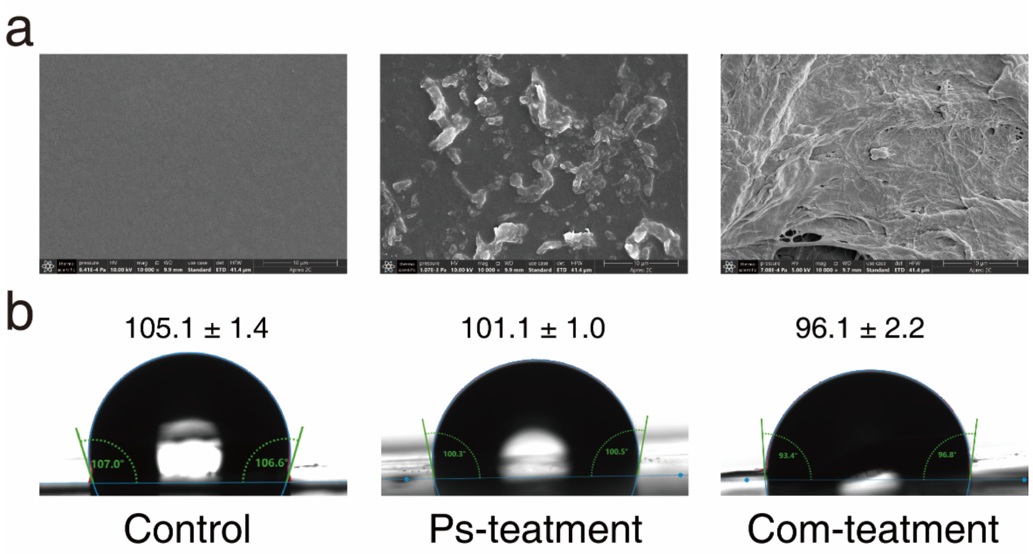

Figure S2. Degradation evidence of St and the SynCom incubated with PE for 30 days. (a) PE surface changes after 30 days of incubation with St and the SynCom. (b) Water contact angle experiment on PE plastic treated with bacteria.

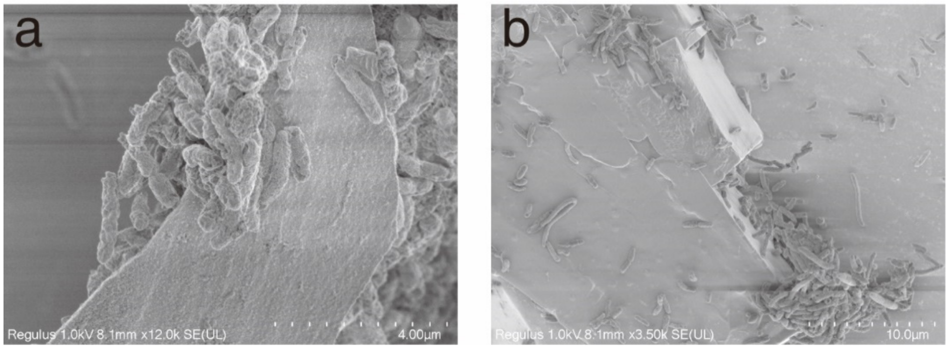

Figure S3. SEM analysis of surface bacteria during PE plastic degradation. (a) SEM images of St on PE surface. (b) SEM images of the SynCom on PE surface.

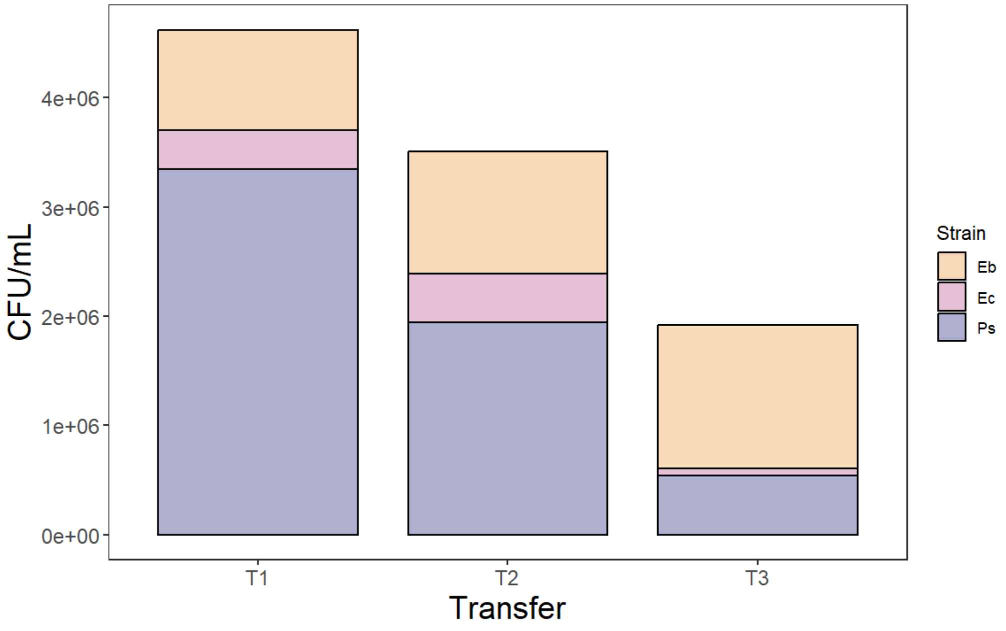

Figure S4. Biofilm formation on PE plastic after the disruption of biofilm structure of microbial community.

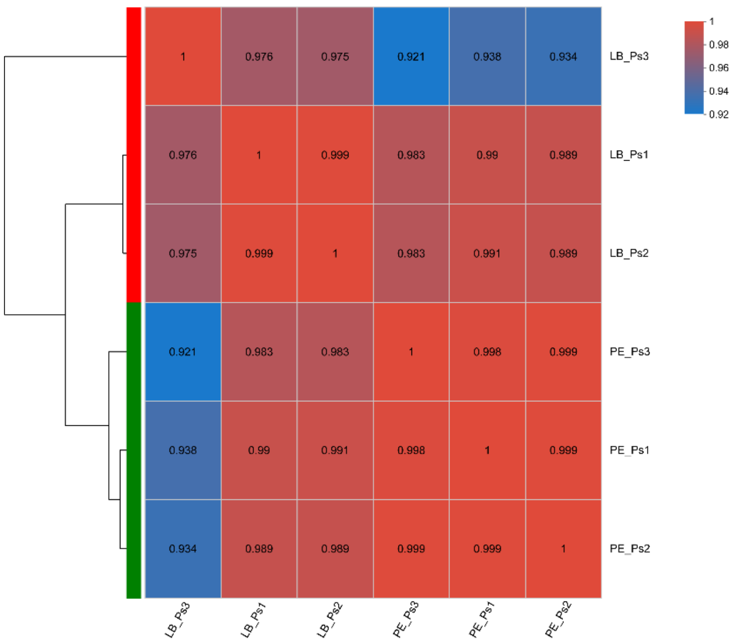

Figure S5. Correlation analysis of expressed genes between samples in PE and LB treatment.

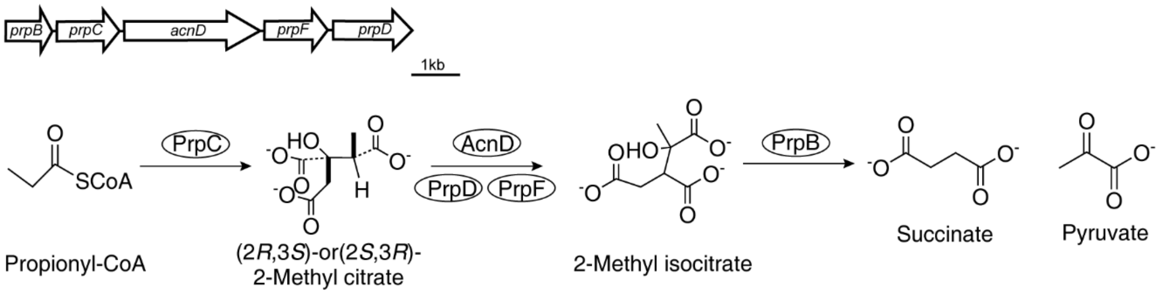

Figure S6. ***prp*** **operon in** ***Pseudomonas spp*****. and propionyl-CoA metabolism pathways.**

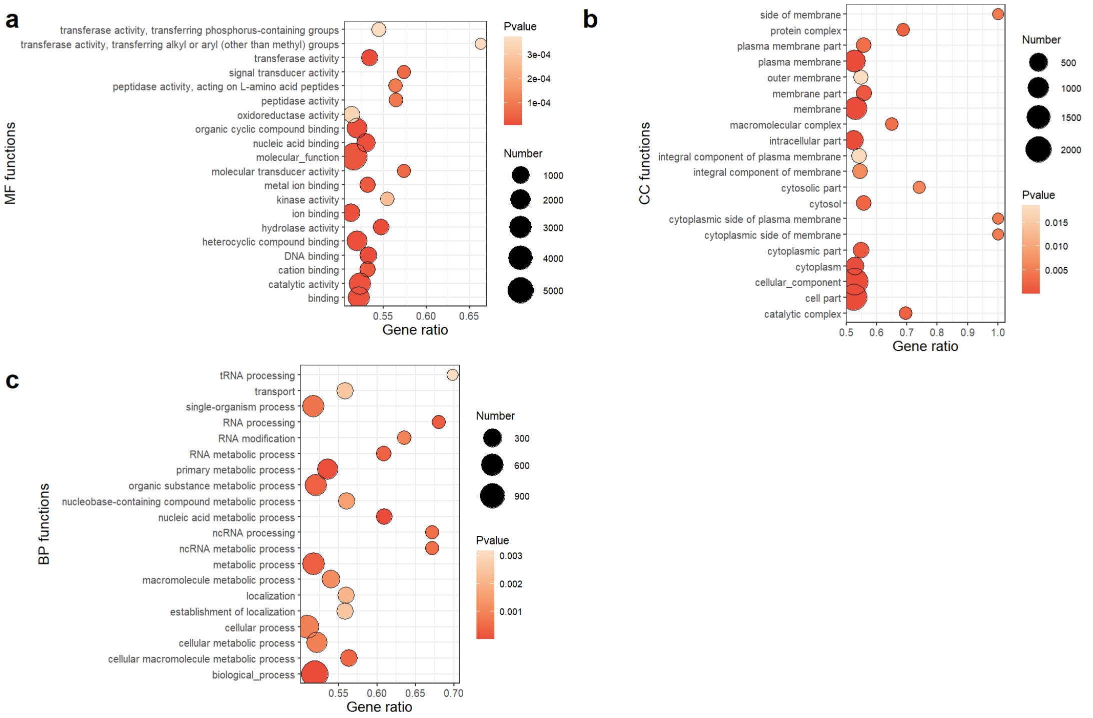

Figure S7. GO enrichment analysis of significantly upregulated genes in the SynCom during cultivation in PE compared to LB medium. (a) Molecular functions. (b) Cellular components. (c) Biological processes.

Supplementary table

Table S1 The primer sets for the relevant 16S rRNA gene amplifications.

Primers	Forward (5'-3')	Reverse (5'-3')
Specific primer for *Stutzerimonas stutzeri* [1]	GTGGGGGACAACGTTTC	TCAGTGTCAGTATTAGC
Specific primer for *Enterobacter cancerogenus* [2]	GCTYTGCTGACGAGTGGCGG	ATCTCTGCAGGATTCTCT GG
Specific primer for *Enterococcus casseliflavus* [3]	GGAAGAAAGTTGAAAGGC	TTAAGAAACCGCCTGCGC

1. Sikorski J, Teschner N, Wackernagel W. Highly different levels of natural transformation are associated with genomic subgroups within a local population of *Pseudomonas stutzeri* from soil. *Appl Environ Microbiol* 2002; **68**: 865–873. doi:10.1128/AEM.68.2.865-873.2002
2. Lehner A, Tasara T, Stephan R. 16S rRNA gene based analysis of *Enterobacter sakazakii* strains from different sources and development of a PCR assay for identification. *BMC Microbiol* 2004; **4**: 43. doi:10.1186/1471-2180-4-43
3. Ryu H, Henson M, Elk M *et al.* Development of quantitative PCR assays targeting the 16S rRNA genes of *Enterococcus* spp. and their application to the identification of *Enterococcus* species in environmental samples. *Appl Environ Microbiol* 2013; **79**: 196–204. doi:10.1128/AEM.02802-12
